# Targeting Parenting Quality to Reduce Early Life Adversity Impacts on Lifespan Cardiometabolic Risk

**DOI:** 10.3389/fpsyg.2021.678946

**Published:** 2021-06-03

**Authors:** Maria E. Bleil, Susan J. Spieker, Cathryn Booth-LaForce

**Affiliations:** Child, Family, and Population Health Nursing, University of Washington, Seattle, WA, United States

**Keywords:** early life adversity, parenting, parenting quality, parental sensitivity, attachment, cardiometabolic health, cardiometabolic risk factors, early life intervention

## Abstract

Mounting evidence that early life adversity (ELA) exposures confer risk for cardiometabolic disease over the lifespan motivated this narrative review to examine parenting quality as a potential intervention target to reduce ELA exposures or mitigate their impact as a way of reducing or preventing cardiometabolic disease. We describe findings from the limited number of family-based intervention studies in ELA-exposed children that have tested parenting impacts on cardiometabolic health outcomes. We then describe the implications of this work and make recommendations for future research that will move this field forward.

## Introduction

Mounting evidence points to the toxic role of early life adversity (ELA) exposures such as family turmoil, stressful or traumatic events, and contextual stressors (i.e., poverty) in shaping cardiometabolic risk over the lifespan. In this context, it is imperative that we build our knowledge of potential interventions to either target the reduction of ELA exposures or to mitigate their impact as a means of reducing or preventing cardiometabolic disease. Given the large number of ELA exposures that have been identified as well as the intractable nature of their effects, much work remains to identify the type and delivery of interventions that would be most effective. In this narrative review, we examine the early emergence of cardiometabolic risk in childhood and the evidence relating ELA exposures to cardiometabolic health over the lifespan. We then provide a conceptual framework using attachment theory to highlight the potential role of parenting quality in mitigating ELA impacts on cardiometabolic health. Finally, we describe findings from the limited number of family-based intervention studies among ELA-exposed children that have tested parenting effects on cardiometabolic health outcomes and make recommendations for future research to move this field forward.

## Early Life Adversity and the Origins of Adulthood Cardiometabolic Disease

Risk for adulthood cardiometabolic disease begins early in life (Olson et al., 2017). Elevated blood pressure and hypertension have been increasing in children (Din-Dzietham et al., 2007; Rosner et al., 2013; Flynn et al., 2017) as have overweight and obesity, with severe obesity in particular rising sharply in children ages 2–5 (Skinner et al., 2018). Type 2 diabetes increased 30% over an 8-year period in one study of almost 2 million children and by 7.1% annually in another study of 4.5 million children (Dabelea et al., 2014; Mayer-Davis et al., 2017). In a community sample, 5% of children ages 7–9 years met criteria for metabolic syndrome and 45% exhibited 1–2 metabolic syndrome components (Dubose et al., 2006). Moreover, insulin resistance and cardiovascular risk factors, including hypertensive status, low HDL cholesterol, and high triglycerides, have been reported in overweight or obese children as young as ages 3–5 years (Bocca et al., 2013). Evidence also shows that children of minority race/ethnic backgrounds exhibit disproportionate risk across these health indicators, including higher rates of hypertension, obesity, and type 2 diabetes compared to their non-Hispanic, white counterparts (Dabelea et al., 2014; Kit et al., 2015; Mayer-Davis et al., 2017; Skinner et al., 2018).

Taken together, the early emergence of risk for adulthood cardiometabolic disease highlights the need to identify modifiable risk factors early in life. ELA exposures are a subset of risk factors or social determinants of health that may be targeted to this end. These exposures encompass a range of experiences that threaten a child's physical or emotional security, such as family dysfunction, stressful or traumatic events, and contextual factors such as socioeconomic disadvantage. ELA exposures are hypothesized to become biologically embedded, possibly through stress-related physiological disruptions in neuroendocrine, immune, and metabolic systems that predispose children to preclinical disease processes and resulting poor cardiometabolic health (Berens et al., 2017). The link between ELA exposures and health was revealed in early findings from The Adverse Childhood Experiences (ACE) Study in which a graded relationship between the number of ELA exposure categories (e.g., physical abuse) and the occurrence of adulthood diseases was observed (Felitti et al., 1998).

To date, a robust and growing literature has substantiated strong and often prospective links between ELA exposures and adulthood health (Galobardes et al., 2008; Shonkoff and Garner, 2012; Su et al., 2015a; Basu et al., 2017; Elsenburg et al., 2017; Suglia et al., 2018), including all-cause and disease-specific mortality (Claussen et al., 2003; Lawlor et al., 2006; Naess et al., 2007), clinical and subclinical cardiovascular disease (CVD) (Roy et al., 2010; Rich-Edwards et al., 2012; Campbell et al., 2016; Hakulinen et al., 2016), CVD risk factors (Danese et al., 2007; Rich-Edwards et al., 2010; Alastalo et al., 2013; Midei et al., 2013; Su et al., 2015b), a worsening of CVD risk over time (Su et al., 2015b; Hakulinen et al., 2016), and negative health behaviors (e.g., cigarette smoking, poor diet) (Anda et al., 1999; Gavrieli et al., 2015). ELA exposures are common in the population, with 60% of adults reporting having experienced at least one type of adversity (CDC, 2010; Bethell et al., 2014). ELA exposures also disproportionately affect vulnerable groups (Slopen et al., 2016; Turney and Wildeman, 2017), suggesting they may contribute to pronounced socioeconomic- and race/ethnicity-based disparities in cardiometabolic health (Chen et al., 2006; Shonkoff et al., 2009).

Although studies of ELA exposures have primarily focused on adulthood health, emerging evidence supports links between ELA exposures and parallel health outcomes in children and adolescents. ELA exposures have been associated with higher systolic blood pressure in children age 5–6 (Smarius et al., 2018) and more rapid increases in blood pressure in young adults (Su et al., 2015b). ELA exposures have also been associated with obesity (Suglia et al., 2012), insulin resistance (Goodman et al., 2007), arterial stiffness (Klassen et al., 2016), and inflammation (Miller and Chen, 2007, 2010; Ehrlich et al., 2016) in children and adolescents, as well as a range of caregiver-reported health problems, including global ratings of the child's health status and medical problems serious enough to require medical attention or impacting functional outcomes such as missing school (Flaherty et al., 2006, 2013; Luby et al., 2017). Findings across the developmental spectrum suggest there is continuity in the role ELA exposures may play in conferring risk for poor cardiometabolic health over periods of childhood, adolescence, and adulthood.

## Parenting Quality, Child Self-Regulation, and Child Cardiometabolic Health

The ELA-health literature is large in volume, but has several gaps outlined in recent publications, including a Scientific Statement from the American Heart Association and a report from an expert panel assembled as a part of an NHLBI workshop entitled “Social determinants of health: Early life adversity as a contributor to disparities in cardiovascular diseases” (Suglia et al., 2018, 2020). One gap concerns a lack of studies examining factors that modify ELA risk or that test interventions to lessen ELA impacts on cardiometabolic health. Recommendations for future research emphasize the need to test early life interventions which act on upstream ELA exposures, especially during vulnerable or sensitive developmental periods when intervention efforts may be most effective (Reynolds et al., 2011; Garner, 2013; McLaughlin et al., 2015; Michalopoulos et al., 2017). One potential target for intervention is parenting quality which is hypothesized to have protective effects on child cardiometabolic health by preventing or lessening the negative impacts of ELA exposures.

Attachment theory provides a potent conceptual framework for considering how parenting quality may “get into the body” to protect child health. Attachment theory posits that the ability of the primary caregiver to appropriately respond to the needs of the child, especially in times of distress, facilitates the development of self-regulatory processes in the child (Ainsworth et al., 1978; Coan, 2016). Self-regulatory processes represent key inter-related pathways through which parenting may operate to influence child health, including, but not limited to, self-regulation of socioemotional well-being (Cooke et al., 2019), stress responses systems (Gunnar, 2017), and emerging health behaviors (Bergmeier et al., 2020). In the context of ELA exposures, enhancing parenting quality may be especially important, potentially reducing the ELA exposures themselves, due to the parent's heighted awareness of their impact, or buffering the harmful impacts of ELA exposures. Moreover, effects of parenting-focused approaches may be far-reaching by generalizing to a broad array of family situations over time and by protecting children against a variety of ELA exposures, many of which are unavoidable.

The role of parenting quality, parent-child relationships, and self-regulatory child behaviors have generally been understudied in relation to physical health outcomes in children. This gap was noted in a recent review in which Bergmeier et al. (2020) urged that the quality of early parent-child relationships, well-studied in relation to areas of child socioemotional development, be examined in relation to childhood weight gain and obesity risk. In longitudinal studies, insecure mother-child attachment as well as a composite of insecure mother-child attachment and reduced maternal sensitivity predicted greater obesity risk in childhood (age 4.5 years) (Anderson and Whitaker, 2011) and adolescence (age 15 years) (Anderson et al., 2012), respectively. Insecure mother-child attachment among preadolescents also predicted changes in maladaptive eating behaviors, including increases in dietary constraint and body image concerns as well as increases in BMI over a 1-year period (Goossens et al., 2012). In one of the few studies with a broader health focus, insecure mother-child attachment assessed in infancy predicted an increased likelihood of having a physical illness in adulthood 30 years later, albeit as assessed by self-reports of health conditions (Puig et al., 2013).

Children with insecure (vs. secure) attachment relationships exhibit poor self-regulation, including maladaptive coping strategies in response to feelings of distress that paradoxically result in unresolved or further distress (Aldao et al., 2010; Cooke et al., 2019). This cyclical process may explain how self-regulatory behaviors, shaped by early parenting practices, influence emerging health behaviors and associated health outcomes. For example, poor self-regulation has been related to problematic eating behaviors (Stoeckel et al., 2017) such as emotional eating, decreased sensitivity to satiety (vs. external) food cues, and binge eating (Frankel et al., 2012; Braden et al., 2014; Dingemans et al., 2017). Emotional eating refers to the use of food as a way to manage negative emotions, potentially serving as a substitute for a lack of support in other areas, including parental support (Haedt-Matt and Keel, 2011). A separate literature shows *adulthood* attachment insecurity is also related to unhealthy eating behaviors (Faber et al., 2018) and eating disorders (Ringer and Crittenden, 2007), as well as to cardiometabolic risk, indexed by metabolic syndrome (Davis et al., 2014; Farrell et al., 2019).

In future studies, more work is necessary to extend these findings to consider physical health outcomes in children beyond those related to eating behaviors and to bridge the child and adulthood attachment literatures in relation to health by examining types of insecure childhood attachment strategies (both minimizing negative emotional expression, Type A, and maximizing negative emotional expression, Type C) (Ringer and Crittenden, 2007; Kozlowska et al., 2011; Crittenden, 2016; Cooke et al., 2019) in relation to parameters of cardiometabolic health in adulthood. In addition, although a significant role for fathers, apart from mothers, is supported by studies relating paternal sensitivity and attachment to child socioemotional and behavioral outcomes (Lucassen et al., 2011; Bureau et al., 2017; Fernandes et al., 2020), father-child attachment has not been examined in relation to child health. Consideration should also be given to the role of grandmothers who play an important role in supporting the parenting behaviors of young mothers and who often contribute to co-parenting efforts, especially in racial/ethnic minority families in whom multigenerational households are more common (Oberlander et al., 2007; Sellers et al., 2011; Cohn and Passel, 2018).

## Conceptual Model: ELA, Parenting Quality, and Child Cardiometabolic Health

**Figure 1:**
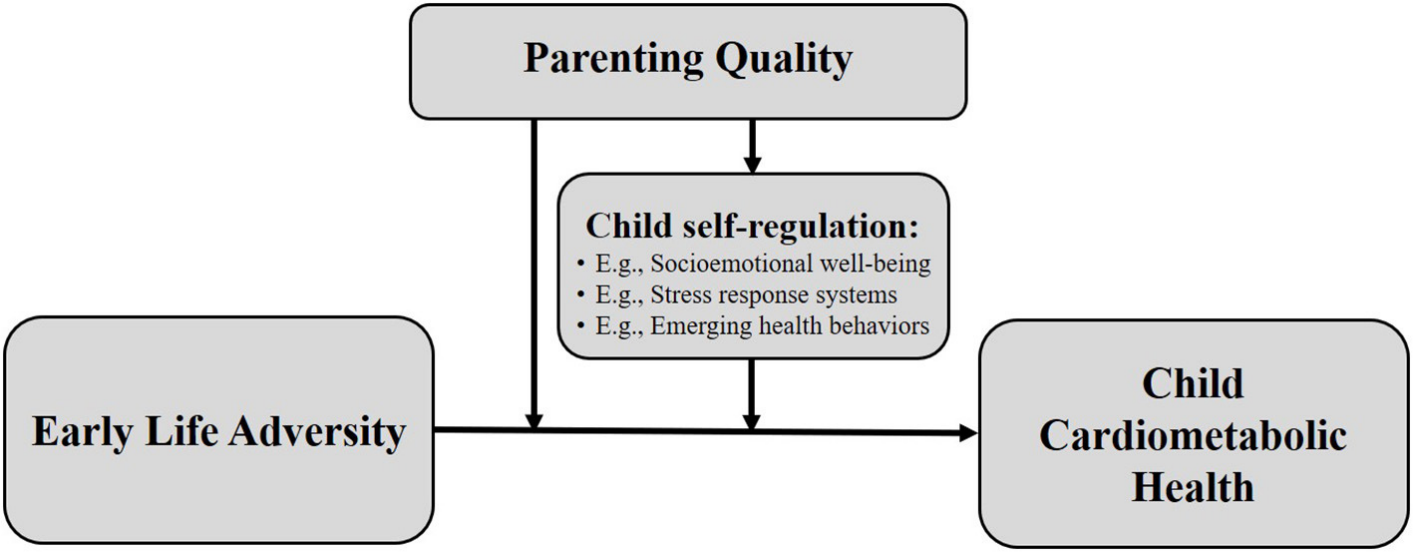
Conceptual model depicting hypothesized role of parenting quality as a protective factor in reducing early life adversity exposures or in mitigating their impact on child cardiometabolic health, in part, through child self-regulation mechanisms.

Based on the findings described above, a conceptual model is presented in Figure 1, depicting the hypothesized role of parenting quality as a protective factor in reducing ELA exposures or in mitigating their impact on child cardiometabolic health, in part, through child self-regulation in areas of socioemotional well-being, stress regulation, and the emergence of early feeding and sleep practices. In this framework, it is plausible that interventions to improve parenting quality among ELA-exposed children may reduce or even prevent cardiometabolic disease. Targeted areas for risk reduction may include chronic inflammation, individual cardiometabolic risk factors, and health behaviors. Although not an exhaustive list, these risk markers are important areas of focus as they are influenced by ELA exposures, show meaningful variation in children, and predict future clinical cardiometabolic disease.

Specifically, levels of chronic inflammation are elevated in individuals with disadvantaged backgrounds including histories of poverty, child abuse, and family stress (Taylor et al., 2006; Danese et al., 2007; Baumeister et al., 2016). Chronic inflammation is itself correlated with obesity in children (Shashaj et al., 2014) and predicts the development of cardiometabolic diseases (e.g., type 2 diabetes) (Ridker et al., 2000a,b; Pradhan et al., 2001; Pradhan and Ridker, 2002)—some emerging even in early childhood (Hernandez et al., 1998; Weiss, 2007; Messiah et al., 2012; Bocca et al., 2013; Shashaj et al., 2014; Bornhorst et al., 2016; Perng et al., 2016). Likewise, cardiometabolic risk factors, risk factor composites such as metabolic syndrome, and relevant health behaviors are all negatively impacted by ELA exposures (Berens et al., 2017). The early emergence of cardiometabolic risk factors, including, for example, patterns of weight gain in the first years of life, predict trajectories of obesity and cardiometabolic risk over time (Stettler et al., 2003). Thus, there is strong support for the proposed conceptual model describing directional associations between ELA, parenting, and child cardiometabolic health that may ultimately lead to better understanding adulthood risk for cardiometabolic disease.

In future studies, important elaborations on this model will be necessary, including consideration of the developmental context of ELA exposures that might heighten risk for cardiometabolic disease. The Developmental Origins of Health and Disease Hypothesis, for example, focuses on exposures during the period of gestation in relation to adulthood cardiovascular risk (Barker et al., 1990; Barker, 1992). Another developmental period of interest is puberty during which time the occurrence of ELA exposures may also confer excess cardiovascular risk (Bleil et al., 2015). Furthermore, the pathway of pubertal development itself is of interest as ELA exposures appear to accelerate the onset of puberty (Belsky et al., 1991; Moffitt et al., 1992; Ellis, 2004; Bleil et al., 2013, 2021) and earlier pubertal timing, in turn, predicts post-pubertal weight gain, worsening CVD risk factor profiles, and incident cardiometabolic disease, and early mortality (Cooper et al., 1999; Frontini et al., 2003; Feng et al., 2008; Jacobsen et al., 2009; Lakshman et al., 2009).

## Parenting Quality: Evidence From Observational and Intervention Studies

### Parenting as a Moderator

A growing number of observational studies have examined the potential moderating role of parenting quality. Findings show responsive caregiving buffers impacts of ELA exposures on a range of health outcomes in adults and children (Evans et al., 2007; Chen et al., 2011; Miller et al., 2011; Asok et al., 2013; Carroll et al., 2013; Farrell et al., 2017; Bernard et al., 2019a). In one study, adults who were socioeconomically disadvantaged as children, but experienced high levels of maternal warmth, displayed fewer pro-inflammatory risk markers compared to their adult counterparts in whom maternal warmth was low (Chen et al., 2011). In another study, consistency in the quality and timing of parent-child interactions was linked prospectively to healthier *in vitro* inflammatory responses in adolescence (Manczak et al., 2018). Findings extend beyond the examination of inflammatory outcomes as well, showing responsive caregiving also buffers impacts of ELA exposures on metabolic syndrome, allostatic load, and BMI, as well as self-reports of physical symptoms and self-ratings of health (Evans et al., 2007; Miller et al., 2011; Carroll et al., 2013; Farrell et al., 2017).

### Parenting Focused Interventions

With respect to randomized controlled trials, few studies have tested family-based interventions among ELA-exposed children to determine whether improvements in parenting may attenuate ELA impacts on cardiometabolic health. Findings among these studies, however, warrant special attention to evaluate evidence for the role of parenting, the ways in which parenting may be leveraged in future studies of child cardiometabolic health, and the broader implications for action in clinical and policy-making settings.

In the Strong African American Families (SAAF) study of vulnerable, low-income families, mothers and their children (11 years old) were randomized to a multifaceted family-based intervention versus control condition in which only the study assessments were administered (Brody et al., 2004). In secondary analyses of a subset of the mother-child dyads, children who received the intervention were found to have lower levels of inflammation 8 years later, as reflected by a composite of markers (IFN-γ, IL-10, IL-1β, IL-6, IL-8, and TNF-α) (Miller et al., 2014). Parenting quality, one of the multiple targets of the intervention, mediated this effect; intervention-related improvements in parenting, including *both* increases in nurturant-involved parenting and decreases in harsh-inconsistent parenting were associated with the lowest levels of inflammation. Moreover, improvements in parenting were greatest, and levels of inflammation the lowest, among the most at-risk families, suggesting the most vulnerable families experienced the most intervention benefit.

Additional secondary analyses in the SAAF study showed intervention effects extended to other cardiometabolic risk indicators as well, including metabolic syndrome and pre-diabetes (Brody et al., 2017, 2019; Chen et al., 2017, 2018). Randomization to the family-based intervention (vs. control condition) mitigated impacts of unsupportive parenting on metabolic syndrome assessed at age 25 with mediational analyses showing effects were attributable to changes in the intervention targets pertaining to parenting quality and parent-child relationships (Chen et al., 2018). Similarly, the family-based intervention (vs. control condition) was found to lessen risk for higher fasting glucose, a marker of pre-diabetes, at age 25 and to eliminate the association between the number of adversity exposures and pre-diabetes (Brody et al., 2017).

In another family-based intervention study, children and their parents who had a history of involvement with child protective services (CPS) due to an identified risk in the home (e.g., maltreatment, domestic violence) were randomized to receive the Attachment and Biobehavioral Catch-up (ABC) intervention vs. control condition in which educational materials about child development were delivered (Bernard et al., 2019a). The ABC intervention used parent coaching and feedback to promote parents' responsivity to child distress as a way of enhancing attachment security and child self-regulation. In the ABC intervention group, 52% of the children were classified as securely attached vs. 32% in the control condition, and attachment security predicted lower BMI at age 4 years and steeper declines in BMI between ages 2 and 4 years. While there was no direct effect of the ABC intervention on child BMI, the mediated or indirect effect of the intervention via attachment security reached marginal significance. In a separate analysis of a subset of children drawn from the same study, children with insecure (vs. secure) attachments exhibited higher levels of inflammation which predicted increases in BMI between ages 4 and 8 years, highlighting the potential mechanistic role of inflammation (Bernard et al., 2019b).

## Implications and Future Directions

The studies reviewed above, describing effects of family-based interventions on health outcomes, highlight the important role of parenting as the “key ingredient” in such interventions. Findings indicate that the influence of parenting on these outcomes may even be causal as suggested by randomized control trials involving families randomized to receive parenting-focused training vs. control conditions. In this context, the use of parenting support interventions as a tool to improve cardiometabolic health in children is an exciting and promising new direction. Importantly, evidence also shows that intervention effects are stronger in families in greatest need (Miller et al., 2014), raising the possibility that such interventions may attenuate pronounced ELA-related health burdens in the most vulnerable families.

As a next step, it is important to move this literature forward by focusing on parenting quality directly as well as the potential mechanisms of its effects (e.g., improved child self-regulation). There is immense untapped potential in existing evidence-based parenting interventions that have already been developed to enhance outcomes in areas of child socioemotional well-being and parent-child relationships. These same interventions should be re-examined in relation to child health outcomes. Some examples of established parenting interventions include the ABC intervention mentioned above (Bernard et al., 2019a) and the Promoting First Relationships program, an attachment theory based program which focuses on enhancing positive parent-child relationships (Kelly et al., 2008). Another intervention, the New Beginnings Program, is a parenting-focused program designed to prevent problems in children who experience adversity such as parental divorce (Sandler et al., 2020). Thus, an urgent research question concerns whether existing parenting interventions that are effective in improving child socioemotional well-being and parent-child relationship outcomes, are also effective in reducing ELA impacts on child cardiometabolic health.

Existing parenting interventions are also uniquely positioned for large-scale dissemination through mechanisms such as the federally funded Maternal, Infant, and Early Childhood Home Visiting (MIECHV) program or other established home-visiting programs. MIECHV, described here to illustrate this potential, started in 2010 as a provision within the Affordable Care Act to provide states with resources for home visiting. Seventy-five percent of funds to the states are required to go to evidence-based home visiting programs. Currently, there are 18 programs listed as evidence-based (“US Department of Health and Human Services, Home Visiting Evidence of Effectiveness^1^,”). Although a few of these programs have reported at least some effects on “positive parenting” and “child health” defined broadly, none have tested the hypothesis presented here—that enhanced parenting quality may prevent or mitigate ELA impacts on child cardiometabolic health. It is plausible that if the benefits of one of these programs were found to extend to child health, there may be “shovel ready” treatment options available for dissemination on a large scale. Dissemination through a mechanism such as MIECHV would make it possible to reach thousands of mother-infant dyads during a sensitive developmental period that sets the stage for lifespan cardiometabolic risk.

## Conclusions

In response to mounting evidence that ELA exposures confer risk for cardiometabolic disease, starting even in early childhood, we highlighted the role of parenting as a potential focus of intervention for the reduction of ELA exposures or their mitigation as a way of reducing or preventing cardiometabolic disease. Among the limited number of family-based intervention studies that examined cardiometabolic health outcomes in ELA-exposed children, significant intervention effects on health outcomes were mediated by improvements in parenting behaviors specifically, confirming the key role of parenting. These findings raise the profile of existing evidence-based parenting interventions that have primarily focused on child socioemotional and behavioral outcomes by identifying the opportunity to test these interventions in relation to child cardiometabolic health. Moreover, existing parenting interventions, if found to also benefit child health, have the potential for broad dissemination through home-visiting mechanisms such as the federally funded MIECHV program, making it plausible to reach the most vulnerable families in whom ELA exposures and cardiometabolic risk factors are disproportionately prevalent.

## Author Contributions

MB developed the study concept and drafted the manuscript. SS and CB-L provided critical revisions. All authors read and approved the final version of the manuscript for submission.

## Conflict of Interest

The authors declare that the research was conducted in the absence of any commercial or financial relationships that could be construed as a potential conflict of interest.

## Footnotes

^1^US Department of Health and Human Services, Home Visiting Evidence of Effectiveness. *Evidence-Based Models Eligible to Maternal, Infant, and Early Childhood Home Visiting (MIECHV) Grantees*. Retrieved from: https://homvee.acf.hhs.gov/HRSA-Models-Eligible-MIECHV-Grantees.

## Abbreviations

ABC: Attachment and Biobehavioral Catch-up
BMI: body mass index
CVD: cardiovascular disease
ELA: early life adversity
MIECHV: Maternal, Infant, and Early Childhood Home Visiting
SAAF: Strong African American Families.
